# Evaluation of a Direct-Instruction Intervention to Improve Movement and Preliteracy Skills among Young Children: A Within-Subject Repeated-Measures Design

**DOI:** 10.3389/fped.2017.00298

**Published:** 2018-01-17

**Authors:** Chloe Bedard, Emily Bremer, Wenonah Campbell, John Cairney

**Affiliations:** ^1^INfant and Child Health (INCH) Lab, Department of Health Research Methods, Evidence, and Impact, McMaster University, Hamilton, ON, Canada; ^2^INfant and Child Health (INCH) Lab, Department of Kinesiology, McMaster University, Hamilton, ON, Canada; ^3^INfant and Child Health (INCH) Lab, School of Rehabilitation Science, McMaster University, Hamilton, ON, Canada; ^4^Faculty of Kinesiology and Physical Education, University of Toronto, Toronto, ON, Canada

**Keywords:** direct-instruction, child development, school readiness, fundamental movement skills, emergent literacy, early intervention

## Abstract

**Objective:**

School readiness involves the development of foundational skills such as emergent literacy and fundamental movement skills as well as the capacity to attentively engage in instructional situations. Children do not develop these skills naturally; therefore, they need the opportunity to develop these skills in their early years prior to entering school. The objective of the current study was to evaluate the effectiveness and feasibility of a direct-instruction movement and preliteracy intervention in children aged 3–4 years.

**Methods:**

A within-subject repeated-measures design, embedded within a wait-list control study, was used to evaluate the intervention. The intervention was run across 10 weeks with 1 h weekly sessions. Each weekly session consisted of 30-min of movement skill instruction (e.g., through single-step acquisition strategies), 15-min of free play during which time children had access to a variety of equipment (e.g., balls, hula hoops, etc.) or toys (e.g., puzzles, building blocks), and a 15-min interactive reading circle during which children read a storybook and were taught 1–2 preliteracy skills (e.g., alphabet knowledge, narrative knowledge, etc.). A convenience sample of 11 children (mean age = 45.6 months, SD = 7.3) was recruited. All children were assessed four times: baseline (Time 1), pre-intervention (Time 2), post-intervention (Time 3), and 5-week follow-up (Time 4). Gross motor skills and preliteracy skills were assessed at each time point.

**Results:**

There was a statistically significant effect of time on the change in gross motor skills (Wilks’ lambda = 0.09, *p* = .002), print-concept skills (Wilks’ lambda = 0.09, *p* = .001), and alphabet knowledge (Wilks’ lambda = 0.29, *p* = .046). *Post hoc* analyses reveal non-significant changes between time 1 and 2 for motor and print-concept skills and significant changes in all three outcomes between time 2 and time 3.

**Conclusion:**

Participation in a direct-instruction movement and preliteracy program led to positive improvements in gross motor skills, print-concept knowledge, and alphabet knowledge in 3- to 4-year-old children over time. Future research needs to evaluate the effectiveness of this movement and preliteracy skill intervention on various other indicators of child development and health.

**Clinical Trial Registration:**

Play and Pre-Literacy among Young Children (PLAY) NCT02432443.

## Introduction

The definition of school readiness differs depending on one’s theoretical perspective; however, contemporary developmentalists agree that it is multifaceted and involves readiness of both the child and their environment to receive all available benefits conferred in the school setting. Readiness skills at the level of the child include development in several areas, such as cognitive, socio-emotional, and motor domains. School readiness at the level of the environment includes, but is not limited to, provision of quality community-based programs, professional development of early childhood education teachers, and supporting parental capacity to help their children grow and develop (1). The degree of readiness is dependent on the proficiency level of children in a number of important intellectual and developmental domains, such as movement ability and emergent literacy skills, among others (2). It is critical that the foundations of these domains are laid before entering school to prepare children for further growth in these areas and facilitate their success in the development of new and complex skills (3).

Two important child-level school readiness skills are movement and preliteracy skills. The importance and relevancy of preliteracy skills for school readiness has been demonstrated extensively. Preliteracy skills include an understanding of print knowledge (e.g., being able to distinguish between print and picture), vocabulary, phonological awareness (e.g., knowledge about the individual sounds of spoken words), and narrative knowledge (e.g., understanding how stories are sequenced and described) (4). When explicit attention is drawn to developing these emergent literacy skills in early childhood, children are better prepared for later academic interactions in the classroom (5). With foundational emergent literacy skills, children can develop new knowledge about alphabet principles and skill in word recognition, reading fluency, and comprehension (4). Development of preliteracy skills not only facilitates these later literacy skills but has been shown to enhance social-behavioral and more general academic achievement (5).

Movement skills, specifically gross motor skills, involve whole body movements coordinated by large muscle groups; these skills include walking, running, jumping, hopping, galloping, throwing, and catching. Evidence from both experimental and observational studies show that attainment of these movement skills not only allows children to independently participate in physical activities (6) but also improves brain function (7, 8), social development (9, 10), self-concept (11), and academic achievement (12). Furthermore, children with motor skill deficits tend to demonstrate lower levels of physical activity (13), poorer self-esteem (14), lower levels of cognitive control (15), and poorer social function (16) compared to children with higher movement competence.

Beyond preparation for school entry, the development of movement skills is essential for long-term health and well-being *via* the influence of movement skills on life-long physical activity (6) and the subsequent physical and mental health benefits that accrue to individuals who lead an active lifestyle (17–19). According to the developmental model proposed by Stodden et al., movement skills are bi-directionally related to both perceived motor competence and health-related fitness (20). Through enhancement in actual and perceived movement skills, children will be more able and more motivated to engage in physical activity and thus maintain a healthy physical trajectory (20). There is strong theoretical and empirical support for the development of movement skills as the pivotal determinant that can set an individual on a positive health trajectory (6). Similarly, literacy skills in general are also essential for health, by ensuring individuals can make informed decisions about health care and healthy lifestyle choices (21). In children, early literacy and reading skills also have been positively related to self-regulation (22–24), which itself is related to positive mental and physical well-being later in life (25). In this sense, interventions designed to improve movement and preliteracy skills are foundational not only for school readiness but for long-term health and well-being as well.

Beyond possessing developmental skills (e.g., movement and preliteracy), school readiness includes being able to participate in classroom activities as well as attend and respond to instruction (26). While the strategy of direct and deliberate instruction is common in many preliteracy curricula [e.g., Justice and colleagues (27)—“experimental explicit intervention”], this is not always the case in movement skill programs. However, Robinson and Goodway (11) demonstrated that direct instruction, whether delivered in a low autonomy or a mastery motivational climate, improved the object control skills of preschoolers. Alhassan and colleagues (28) found significant gains in movement skills in preschool children following a direct-instruction movement-based intervention compared to a free-play program. These two experimental studies highlight the importance of using systematic and explicit instruction strategies to enhance skill levels because children do not inherently possess these skills. Furthermore, there is evidence to suggest that teaching both movement skills and preliteracy skills in a single program may have synergistic effects on the gains achieved in both skill domains. For example, Callcott and colleagues (29) taught preschool children movement skills and preliteracy skills simultaneously in their intervention and found gains in movement skill above those achieved by a group of children learning movement skills in isolation. Additionally, an intervention that teaches both movement and preliteracy skills appeals to parents, educators, and children. Parents and teachers are interested in and attentive to ways to help their children and students meet these movement-related and academic goals and children are naturally drawn toward activities like jumping, skipping, and reading story books because they are inherently fun and enjoyable.

The readiness of a child to enter into school also is influenced by the capacity of parents to teach and support their child’s development in these domains. Therefore, it is critical to include parents or caregivers in these direct-instruction programs to provide them with guidance on teaching strategies that can be implemented at home. In fact, this has been the recommendation from research for both movement and preliteracy skill interventions (30, 31). The systematic review by Veldman and colleagues (30) specifically noted the absence of parental or caregiver involvement in movement-based interventions for children beyond sending home educational handouts and subsequently recommended that parents should be actively engaged throughout the program and encouraged to practice the skills at home. Shared book preliteracy interventions have typically included and emphasized the role of parents and caregivers; however, these interventions do not use explicit instruction strategies for skill development (32). Interventions employing direct-instruction strategies are typically delivered by teachers (33) or trained researchers (34) without extension to parents. By excluding parents or caregivers in the delivery of effective preliteracy intervention the frequency and dose of the intervention is inherently limited to time spent with the interventionist; however, parents may have many opportunities to implement these lessons at home if they have been provided with the knowledge and tools to do so.

In summary, both emergent literacy and fundamental movement skills are important for school readiness, and confer long-term health and well-being benefits for children. However, there is a need for systematic evidence-based approaches that target children’s movement and preliteracy skills. At present, best practice suggests that these approaches need to involve direct-instruction teaching strategies with explicit and active involvement with parents or caregivers. Thus, the purpose of this study was to evaluate the change in movement and preliteracy skills following a 10-week direct-instruction movement and preliteracy skill intervention for children aged 3–4 years.

## Materials and Methods

### Design

A within-subject repeated-measures design was used to evaluate the program. The participants originally had been assigned to the wait-list control arm of a quasi-experimental study, the results of which have been published previously (35). However, by continuing to follow and offer these children the same intervention that the experimental group had received, we could assess change in motor skill and preliteracy using a within-subject design. We are unable to combine the results of the original quasi-experimental study with the current results because children in the wait-list control group serve as their own control; therefore, combining all children who receive the intervention and comparing them to the control period in which they did not receive the intervention, would violate the statistical assumption of independent observations.

All children were assessed four times: baseline (Time 1), pre-intervention (Time 2), post-intervention (Time 3), and 5-week follow-up (Time 4). While the results for time 1 and time 2 were previously reported in the study by Bedard et al. (35), time 3 and 4 results have not been reported.

### Participants

A convenience sample of families was recruited through advertisements at local community centers (e.g., Early Years Centres, Boys and Girls Clubs, Public Libraries) from May to July 2015. Children were eligible to participate if they were between the ages of 3 years, 0 months, to 4 years, 11 months at baseline, and must not have been diagnosed with any developmental delay or health condition that would prohibit safe participation in the program.

### Intervention

The program took place in the gymnasium of a local Early Years Centre and was led by two graduate students (EB and CB) with prior experience in implementing movement and preliteracy programs. The program ran once per week for 10 consecutive weeks and each 60-min session consisted of three components: direct movement skill instruction (30 min), unstructured exploratory free play (15 min), and an interactive storybook reading activity (15 min). Key teaching strategies employed throughout all aspects of the program included: an emphasis on the use of correct terminology; individual scaling of skill level; significant and active parent involvement for all aspects of the program other than free play; and the use of a large visual schedule to ease the transition between program activities.

The intervention and its teaching strategies were adapted from a movement skill intervention originally designed for young children with autism spectrum disorder (36, 37) and now used for children with typical development (35). The lesson plans and teaching strategies for the preliteracy component of this intervention were adapted from several evidence-based curricula (31, 38, 39).

#### Direct Instruction for Movement Skills

This first component of the intervention (direct teaching of movement skills) was further divided into four activities: warm-up, two blocks of skill instruction, and an obstacle course. Each week focused on teaching a different movement skill, with the skills progressing in difficulty over the 10-week program (see Table 1 for the Weekly Skill List).

**Table 1:** Weekly skill list.

Week	movement skill	Preliteracy skill
1	Balancing	Pictures vs print
2	Underhand rolling	Characters
3	Leaping and galloping	Pictures vs print
4	Underhand throwing	Setting
5	Jumping	Directional tracking
6	Overhand throwing	Describing the plot
7	Catching	Directional tracking
8	Hopping	Sequencing events
9	Kicking	Alphabet knowledge
10	Striking	Alphabet knowledge

To start the program, the children and their parents formed a circle and participated in warm-up games (e.g., the hokey pokey) for approximately 5 min. This provided an opportunity for the children to become comfortable with one another and ease into the start of the program. Next, two 7-min blocks of direct skill instruction occurred with the skill increasing in difficulty over the two blocks. For example, when teaching the overhand throw, the first block of skill instruction would focus on throwing a large ball overhand with two hands. The second block of skill instruction would then work on teaching the children how to throw a smaller ball overhand with one hand. During the blocks of direct instruction, the program leaders would first demonstrate the skill to the children and their parents, while emphasizing the correct form of the skill. The child-parent dyads were then instructed to spread out in the activity area to practice the skill. This ensured there were numerous repetitions of skill practice and opportunities for skill mastery. For example, while teaching the overhand throw, the child would be instructed to throw the ball to their parent. This would progress on an individual basis to throwing further distances, toward a target, etc. Throughout these activities, the program leaders worked their way around the activity space to check in on the child-parent dyads and provide suggestions for the child to scale the skill level up or down, based on individual need. For instance, a child struggling with the overhand throw would be given pointers on how to make it easier, such as using floor markers to indicate how to stand; likewise, a child who was excelling at throwing may be asked to throw further or at a moving target while still using proper form. As parents became more confident in their own teaching abilities, they were encouraged to help their child scale the skill level on their own. Throughout both blocks of direct skill instruction, an emphasis was placed on having children take their time and using correct processes to complete the task (e.g., to throw overhand, there is a wind-up of the ball up and back, a step with the opposite foot, and follow-through of the throwing arm toward the target), rather than rushing through the skill.

Following the two blocks of skill instruction, an obstacle course was set-up for the children to practice the skills that they learned that day, as well as review previously learned skills in a fun activity. The obstacle course each week consisted of 3–4 skill stations set-up in a square path so that the children started and ended in the same location. Parents completed the obstacle course with their child, either helping them through it, or completing it before or after their child to model or mimic the skills, respectively. Each child was given the opportunity to complete the course 3–4 times before transitioning to the second component of the intervention.

#### Free Play

The second component of the intervention consisted of 15-min of unstructured exploratory, child-directed free play. During this time, children were supervised by one of the program leaders and program volunteers, while the second program leader accompanied parents to a separate location to have them complete weekly questionnaires. Children had the option to play with a variety of equipment (e.g., balls, hula hoops, balance beams, etc.) or toys (e.g., puzzles, building blocks) and were given free choice in what they did for the 15 min. The program leader and volunteers engaged in the activities with the children but were instructed not to suggest or discourage the children’s play, but rather to follow their lead in whatever game they chose. At the end of free play, children were instructed to put away whatever they were playing with and form a circle while the parents re-entered the activity space for the final component of the program.

#### Direct Instruction for Preliteracy Skills—Dialogic Shared Storybook Reading

The third component of the intervention was a 15-min interactive storybook reading circle with direct involvement of the parents. Each week, 1–2 preliteracy skills were introduced and developed using one storybook (see Table 1 for the Weekly Skill List). Each book was read twice over the course of the 10 weeks. Parents and children sat in a circle and each pair were provided with the book. The program leader would begin the reading by first introducing the skill using proper terminology. For example, the leader would explain to the group that in today’s story, “We will learn about characters and settings. Characters are who the story is about and the setting is where the story takes place.” Each lesson would continue by reviewing the picture and words (i.e., title, author, and illustrator) on the front cover of the book. Next, the leader would encourage the parents to follow along during the book reading. Periodically, the leader would ask open-ended questions that helped develop the children’s understanding of the particular preliteracy skill. For example, the leader might ask children “Where are they now?” to highlight how to describe the setting of the story. The leader would repeat and expand children’s responses using appropriate narrative terminology to help strengthen understanding of the skill. Children were encouraged to raise their hands to answer a question and the leader ensured each child had an opportunity to respond to a question. Strategies to simplify the question included providing children with multiple response options, or modeling an appropriate response and asking the child to repeat back the model answer.

#### Take-Home Suggestions

At the end of each session, parents were provided with a 1-page handout outlining the movement and preliteracy skill learned that day. These handouts provided the parents with a description on correct execution of the skill, as well as ideas for games that could be played at home to practice the skill.

### Outcome Measures

#### Demographic and Engagement Survey

A demographic questionnaire was completed at baseline by the child’s parent and included questions about the parent and the child on age, gender, ethnicity, parental education and occupation, and household income. A parent engagement questionnaire was administered at each of the four assessments to assess how frequently the children were engaging in movement and preliteracy activities at home, and the parental uptake of the teaching strategies used in the program.

#### Movement Skills

Children were administered the gross motor subtests of the Peabody Developmental Motor Scales-2 (PDMS-2) (40) at each assessment. The PDMS-2 is a standardized assessment designed to measure the progress of development of gross and fine motor skills in children from birth to age 6. The gross motor subtests—stationary, locomotion, and object manipulation—were administered by two trained graduate students. The sum of the raw scores of each of the three subtests was used as the dependent variable in the primary analysis. The assessment required approximately 30–45 min to conduct (41). The validity and sensitivity to change of the test has been assessed previously in 4-year-old typically developing children and the inter-rater reliability is 0.89 (41, 42).

#### Preliteracy Skills

Children were administered the Preschool Word and Print Awareness (PWPA) test and the Phonological Awareness Literacy Screening: Preschool (PALS-PK) to measure print-concept knowledge and alphabet knowledge (43, 44). The PWPA tests children on their print-concept knowledge, such as print directionality and print function, using 14 items administered in an interactive storybook reading format (43). Raw scores are then transformed into standardized scores with a mean of 100 and SD of 15. The PWPA has strong validity; item reliability is 0.74 and inter-rater reliability is 0.94 in a sample of children aged 3–5 years (45, 46). The PALS-PK Upper-case Alphabet Recognition task involves children naming each of the 26 letters of the alphabet as they are presented in a random order. The inter-rater reliability coefficient of this task is 0.99 (44). These measurements together took approximately 15 min to complete and were administered by a trained graduate student.

#### Attendance and Home Practice

Weekly attendance was taken at the program and the frequency of weekly home practice was measured with a parent-reported questionnaire completed each week during the free-play component of the program.

### Procedure

**Figure 1:**
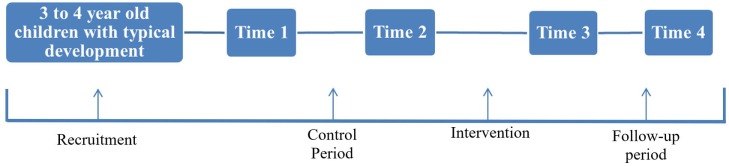
Flow diagram of the study procedure.

The study received ethical approval from the Hamilton Integrated Research Ethics Board at McMaster University. All study appointments took place in a research lab at the university, while the intervention took place at an Early Years Centre in the local community. Study eligibility was confirmed by telephone at which time parents scheduled their first study appointment. Informed written consent was obtained at the first appointment. At each appointment, children were assessed on their movement and preliteracy skills and parents were asked to complete the demographic (at baseline only) and engagement questionnaires. After the first appointment (time 1), families were asked to come back in for their second appointment (pre-intervention) approximately 10 weeks later where they were reassessed on all measures. Within 1–2 weeks of the second appointment, families began to participate in the 10-week intervention. Upon completion of the intervention, families came back to the lab for their third appointment (post-intervention), and subsequently 5–6-weeks later for their follow-up assessment. See Figure 1 for the flow diagram depicting the study procedures.

### Statistical Analyses

Descriptive statistics were computed on the demographic characteristics of the sample, attendance, and at-home practice rates. The primary analyses were three repeated-measures analyses of variance (ANOVA) to assess change in the children’s gross motor skills (raw scores), print-concept knowledge (standardized scores), and alphabet knowledge across all four time points. Least significant difference (LSD) *post hoc t*-tests were applied to models that were statistically significant overall to determine significant changes between specific time points (e.g., T2 vs T3). Secondary analyses included two repeated-measures ANOVAs examining change in parental engagement in both movement and preliteracy activities. A two-tailed alpha value of 0.05 was used to determine statistical significance.

## Results

### Descriptive Characteristics

Eleven families were eligible and consented to participate in the first study appointment. All 11 families participated in the second appointment and 9 (82%) entered into the intervention and completed the remainder of the study. The analytical sample includes 9 children (6 boys) ranging from 36 to 59 months (mean = 45.6, SD = 7.3). Table 2 describes the demographic characteristics of the sample.

**Table 2:** Sample demographic characteristics.

Variable	*N* = 11
Child’s mean age in months (SD)	45.6 (7.3)
Child’s gender%	
Male	55
Child’s ethnicity%	
Black	9
South Asian	9
Mixed ethnicity	9
White	73
Parent age (years)	33.9 (4.1)
Parent education%	
College/technical training	45
University degree	55
Parent income%	
Less than $50,000	36
Greater than $50,000	64

### Intervention Effects

**Figure 2:**
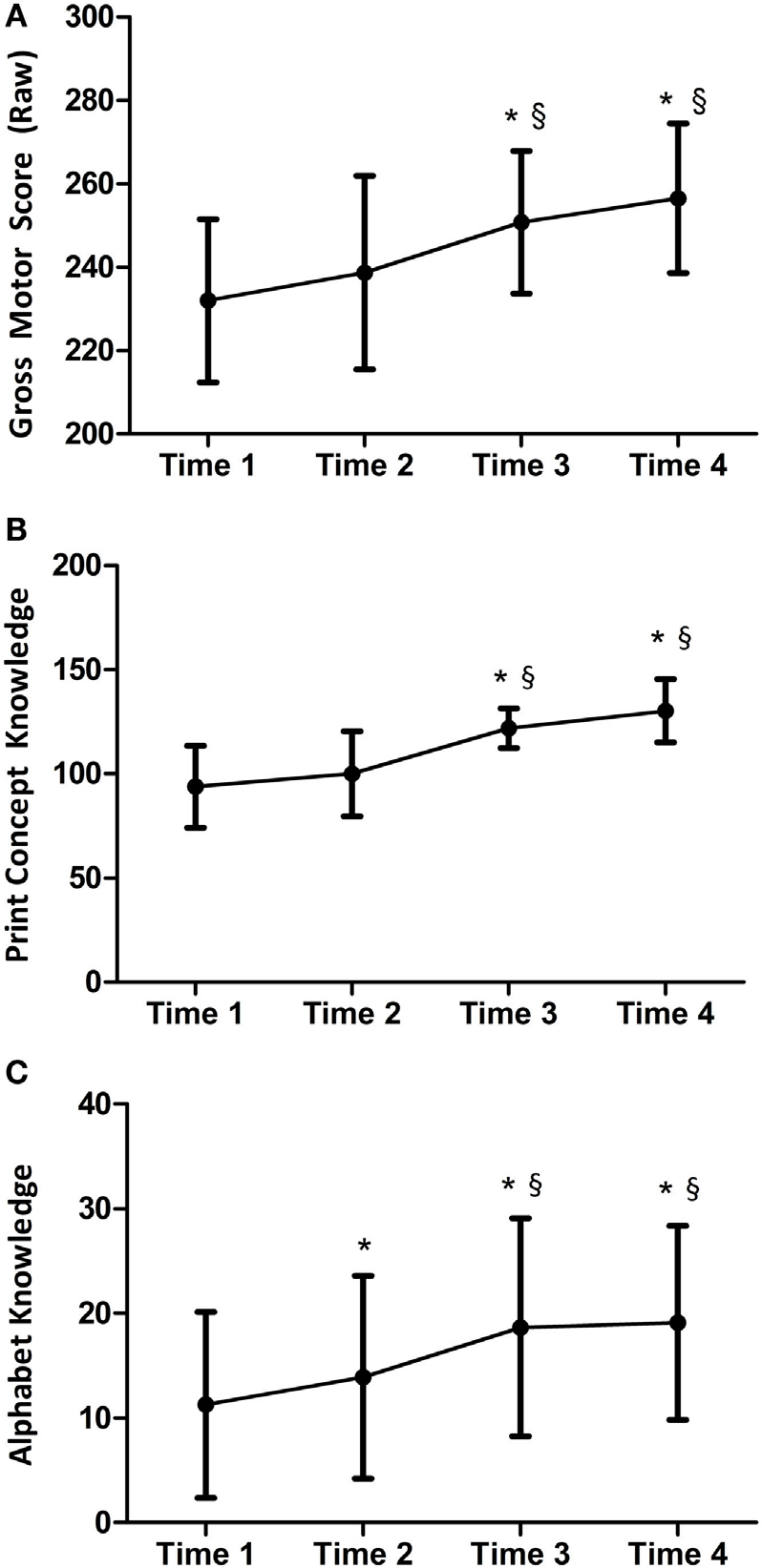
Change over time in the primary outcome measures. **(A)** Gross motor skills; **(B)** print-concept knowledge; and **(C)** alphabet knowledge; *statistically significantly different from time 1; §statistically significantly different from time 2.

The median attendance was 8 of 10 sessions, and the average rate of at-home practice was 48% and 46% for the movement skill and preliteracy activities, respectively. Mean scores and SDs of the primary outcomes (movement and preliteracy skills) at each time point are presented in Table 3 and displayed graphically in Figure 2.

**Table 3:** Scores over time on the primary outcomes.

Time	Gross motor skill (raw)	Print concept knowledge	Alphabet knowledge
1	230.7 (21.2)	93.8 (19.5)	13.1 (8.8)
2	236.0 (24.0)	102.6 (17.7)	16.0 (9.4)
3	250.8 (17.1)	121.9 (9.5)	18.7 (10.4)
4	256.6 (17.9)	130.3 (15.3)	19.1 (9.30)

There was a statistically significant effect of time on the change in gross motor skills (Wilks’ lambda = 0.09, *p* = .002), print-concept skills (Wilks’ lambda = 0.09, *p* = .001), and alphabet knowledge (Wilks’ lambda = 0.29, *p* = .046). For gross motor skills, LSD *post hoc* tests reveal no statistically significant changes between time 1 and 2 (during the control period) and 3 and 4 (follow-up period); however, there was a statistically significant change between time 2 and 3 (during the intervention period; mean difference = 14.8, *p* = .015). *Post hoc* LSD tests for print-concept knowledge revealed no statistically significant changes between time 1 and 2 (during the control period) or time 3 and 4 (follow-up period); but there was a statistically significant change between time 2 and 3 (during the intervention period; mean difference = 28.1, *p* < .001). Alphabet knowledge changed significantly between time 1 and 2 (during the control period; mean difference = 2.9, *p* = .048), time 2 and 3 (during the intervention period; mean difference = 2.7, *p* = .04), but there was no statistically significant changes between time 3 and 4 (follow-up period). The results of the secondary analyses revealed that there was no significant effect of time on parent engagement in movement or preliteracy skills. There were no reported adverse effects of the intervention.

## Discussion

Participation in our movement and preliteracy program led to positive improvements in gross motor skills, print-concept knowledge, and alphabet knowledge in 3- to 4-year-old children over time and these gains were sustained over a 5-week follow-up period. Participants in our study were measured four times to ensure a control, intervention, and follow-up period were captured; significant change occurred only after introduction of the intervention thus supporting the attribution of change to the intervention. Our results are consistent with the results of our quasi-experimental study published previously (35) as well as with the overall evidence supporting the effectiveness of interventions designed to improve movement skills in young children (30) as well as the wealth of evidence in favor of dialogic reading activities compared to passive reading strategies (27, 31, 33, 39, 43).

Furthermore, our results are consistent with extant literature supporting direct-instruction techniques compared to exclusive free-play in the development of movement skills or passive shared book reading activity in the development of preliteracy skills (11, 27, 28, 33, 34, 47). The current study demonstrated the feasibility and effectiveness of using direct-instruction techniques in a structured environment in combination with a short bout of free play to strengthen movement and preliteracy skills in young children. There is a large body of evidence supporting the use of free play in the development of important life skills including creativity, socio-emotional skills, and self-regulation (48, 49). However, free-play alone will not allow the maximal development of physical and academic skills because quality programs require planned instruction, clear goals, demonstrations of skill, opportunity for practice, and appropriate and timely feedback (11, 47, 50, 51). With respect to movement-based interventions, Robinson and colleagues acknowledged the critical need to provide explicit instruction to support the development of fundamental movement skills during early childhood and designed a study to test the effectiveness of such a program against a free-play recess intervention in preschoolers. Over a 9-week intervention period, children participating in the direct-instruction intervention demonstrated superior object manipulation skills compared to their peers in the recess group, and these skill gains were maintained at a 9-week follow-up (11). Within the preliteracy intervention research, there is also a wealth of evidence in support of explicit (direct-instruction) interventions as the best-practice approach as this is viewed as the most efficient method to improve preliteracy skills (27, 52). For example, Justice and colleagues (27) tested the effectiveness of an explicit preliteracy intervention against an adult-child shared book reading activity and re-telling activity and found that while both programs improved preliteracy skills, the direct-instruction program demonstrated larger gains in alphabet knowledge, print awareness, phonological segmentation, and rhyme production. Similarly, Hilbert and Eis (33) compared an explicit intervention developed by Laura Justice and Anita McGinty called *Read It Again Pre-K*! intervention against the usual curriculum in a Head Start program and found gains in picture naming ability. While our current study does not test the comparative effects of a direct-instruction intervention against a passive intervention, our results do provide evidence in support of the effectiveness of these explicit instruction techniques in both movement and preliteracy skill domains.

School readiness is about both the content of skill development as well as the process. Therefore, our intervention placed a large emphasis on teaching skills in a format reflective of a school day: that is, one in which children will need to attend to instructions, attempt the skill, self-regulate and learn from mistakes, persist with repetitions of skill execution, engage socially with teachers and peers, and creatively direct their own activities while being respectful and sensitive toward others. By placing children in this semi-structured environment they were not only able to improve their movement and preliteracy skills, but also were given the opportunity to develop self-regulation, pro-social skills, creativity, and a predilection toward learning—all of which define a child ready for school.

The results of this research have important implications on the promotion of health and well-being given the direct and indirect relationships with movement and emergent literacy skills. Improving the movement skill-set of preschool-aged children presumably enables participation in physical activities, which in turn, supports development of more complex movement abilities. Greater motor proficiency can lead to the strengthening of physical self-concept, which may positively reinforce children’s motivation to engage in physical activities and promote cumulative physical and mental health benefits. A gain in preliteracy skills could plausibly lead to gains in specific aspects of executive function because the learning process itself places demands on specific executive functions (i.e., inhibition, cognitive flexibility) (24). Given the relationship between executive function and mental and physical health outcomes (53), long-term health benefits may possibly result from this indirect impact of a preliteracy intervention on executive functions. However, future research will need to formally evaluate both the short- and long-term impact of this intervention on measures of social skills, self-regulation, executive function, physical activity, self-concept, and other areas of health and development.

### Limitations

Notably, this study is limited by a small sample size, few outcome measures, and lack of free-play control group. Analyses were underpowered to detect changes over time within specific gross motor domains (e.g., object control) or the effect of gender on intervention effectiveness. As well, we are limited by our measures to make definitive conclusions on the impact of our intervention on other aspects of school readiness. While movement and preliteracy skills are important components of school readiness, they are not comprehensive or direct measures of school readiness. The lack of control group implementing an exclusively free-play intervention limits our ability to make head-to-head comparisons between direct-instruction and free-play; however, the goal of the current study was not to evaluate the difference between these two approaches, but was to demonstrate the feasibility and effectiveness of direct-instruction in improving both movement and preliteracy skill levels. Lastly, our restricted outcome measures limit our ability to make conclusions about the effect of the intervention on broad measures of health and development (e.g., physical activity and executive function).

## Conclusion

Future research needs to continue to evaluate the effectiveness of this movement and preliteracy skill intervention on various other indicators of child development, school readiness, and the long-term impact throughout childhood.

## Ethics Statement

This study was carried out in accordance with the recommendations of Hamilton Integrated Research Ethics Board at McMaster University with written informed consent from all subjects. All subjects gave written informed consent in accordance with the Declaration of Helsinki. The protocol was approved by the Hamilton Integrated Research Ethics Board at McMaster University.

## Author Contributions

CB designed the study, coordinated recruitment and data collection, designed the preliteracy component of the intervention and assisted with weekly implementation of the intervention, carried out the data analyses, and drafted the initial manuscript. EB assisted with study design, recruitment and data collection, design of the motor component of the intervention and weekly implementation of the intervention, and revised and approved the final manuscript as submitted. WC assisted with design of the intervention, selection and design of outcome measurements, and reviewed and approved the final manuscript as submitted. JC supervised the design and execution of all phases of the study and revised and approved the final manuscript as submitted. All authors approved the final manuscript as submitted and agreed to be accountable for all aspects of the work.

## Conflict of Interest Statement

The authors declare that the research was conducted in the absence of any commercial or financial relationships that could be construed as a potential conflict of interest.

## Abbreviations

PWPA, Preschool Word and Print Awareness; PALS-PK, Phonological Awareness Literacy Screening: Preschool, PDMS-2, Peabody Developmental Motor Scales—2nd edition; ANOVA, analysis of variance.
